# Caspase-3 knockout attenuates radiation-induced tumor repopulation via impairing the ATM/p53/Cox-2/PGE_2_ pathway in non-small cell lung cancer

**DOI:** 10.18632/aging.103984

**Published:** 2020-11-07

**Authors:** Minghui Zhao, Yiwei Wang, Yucui Zhao, Sijia He, Ruyi Zhao, Yanwei Song, Jin Cheng, Yanping Gong, Jianzhu Xie, Yulan Wang, Binjie Hu, Ling Tian, Qian Huang

**Affiliations:** 1 Cancer Center, Shanghai General Hospital, Shanghai Jiao Tong University School of Medicine, Shanghai 201620, China; 2 Shanghai Key Laboratory for Pancreatic Diseases, Shanghai General Hospital, Shanghai Jiao Tong University School of Medicine, Shanghai 201620, China; 3 Department of Central Laboratory, Shanghai Chest Hospital, Shanghai Jiao Tong University School of Medicine, Shanghai 200030, China

**Keywords:** radiotherapy, non-small cell lung cancer, tumor repopulation, caspase-3, DNA damage response

## Abstract

Radiotherapy is an effective treatment for non-small cell lung cancer (NSCLC). However, irradiated, dying tumor cells generate potent growth stimulatory signals during radiotherapy that promote the repopulation of adjacent surviving tumor cells to cause tumor recurrence. We investigated the function of caspase-3 in NSCLC repopulation after radiotherapy. We found that radiotherapy induced a DNA damage response (DDR), activated caspase-3, and promoted tumor repopulation in NSCLC cells. Unexpectedly, caspase-3 knockout attenuated the ataxia-telangiectasia mutated (ATM)/p53-initiated DDR by decreasing nuclear migration of endonuclease G (EndoG), thereby reducing the growth-promoting effect of irradiated, dying tumor cells. We also identified p53 as a regulator of the Cox-2/PGE_2_ axis and its involvement in caspase-3-induced tumor repopulation after radiotherapy. In addition, injection of caspase-3 knockout NSCLC cells impaired tumor growth in a nude mouse model. Our findings reveal that caspase-3 promotes tumor repopulation in NSCLC cells by activating DDR and the downstream Cox-2/PGE_2_ axis. Thus, caspase-3-induced ATM/p53/Cox-2/PGE_2_ signaling pathway could provide potential therapeutic targets to reduce NSCLC recurrence after radiotherapy.

## INTRODUCTION

With over a million deaths reported worldwide annually, lung cancer ranks among the top causes of cancer-related mortality [1]. According to a study, non-small cell lung cancer (NSCLC) accounts for approximately 85% of all lung cancers [2]. Radiotherapy has remained an effective treatment throughout the continuum of NSCLC care. Despite remarkable advances in the treatment of NSCLC using a combination of surgical techniques and systemic chemotherapy or radiotherapy, it has a dismal prognosis due to resistance to the therapy and local recurrence. Consequently, NSCLC has a median survival of less than a year and a 2-year survival rate of less than 20% [3].

Radiotherapy uses high-energy waves to kill tumor cells and shrink the gross tumor mass. However, the surviving tumor cells can repopulate because they can proliferate during the intervals between the radiotherapy sessions [4, 5]. The possible factors underlying this phenomenon include tumor hypoxia [6], inflammation [7], angiogenesis [8], and tumor stemness [9].

In our previous studies, we demonstrated the involvement of apoptosis in tumor repopulation during radiotherapy [10, 11]. Activated caspase-3 not only executes apoptosis but also promotes the release of several growth factors from irradiated, dying tumor cells that stimulate the proliferation of adjacent living tumor cells [10]. We found that activated caspase-3 cleaved cytosolic calcium-independent phospholipase A_2_ (iPLA_2_) and subsequently increased the production of arachidonic acid (AA), a known precursor of prostaglandin E_2_ (PGE_2_). PGE_2_ is a potent mitotic factor and involved in acute inflammatory responses [12]. We named this counterintuitive caspase-induced tumor repopulation mechanism as the “Phoenix Rising” pathway. Caspase-3 is increasingly becoming recognized as a stimulator of cellular proliferation and carcinogenesis. For instance, we previously reported that caspase-3 in dying glioma cells promoted endothelial cell mitosis by activating the NF-κB/Cox-2/PGE_2_ axis to establish a pro-angiogenic microenvironment that promoted tumor repopulation [11]. Similarly, another study demonstrated that activated caspase-3/7 contributed to self-inflicted DNA double-strand breaks (DSBs), elevating the expression of CD133 in glioma cancer stem cells (CSCs) [13].

Because radiations kill tumor cells by inducing DNA lesions, we investigated if the DNA damage repair pathway participated in tumor repopulation. DNA DSBs can arise from exogenous or endogenous stressors. To repair DNA lesions, cells have evolved a complex network called DNA damage response (DDR). DDR pathways consist of numerous proteins that function as part of cell cycle checkpoints and DNA damage repair. The ataxia-telangiectasia mutated (ATM)/p53 cascade participates in DNA damage repair and is the most commonly activated DDR pathway in response to DSBs or errors occurring during the cell cycle [14, 15]. The sensor kinase ATM is recruited to the damaged sites and autophosphorylated at Ser-1981. Next, the activated ATM directly phosphorylates checkpoint kinase 2 (Chk2) on Thr-68 and p53 on Ser-15. The phosphorylated p53 is resistant to ubiquitination and induces cell cycle arrest, apoptosis, or senescence [15–17]. Irradiated cells use the DDR to repair DNA lesions and recover. Radiotherapy works on the principle that irreparable DNA damage may trigger cell death. Moreover, defects in DDR have been reported to cause genetic instability and drive carcinogenesis [18].

We conducted experiments to study the hypothesis that caspase-3 coordinates with the DDR to induce tumor repopulation during radiotherapy in NSCLC. We found that treatment with ionizing radiations induced DDR and apoptosis by activating apoptotic caspase-3 and the ATM/p53 axis. Unexpectedly, activated p53 increased the production of Cox-2/PGE_2_ in the presence of activated caspase-3 in irradiated NSCLC cells. Further, the production of Cox-2/PGE_2_ was remarkably suppressed in caspase-3 knockout (Casp3 KO) NSCLC cells despite the elevated expression of p53. Overall, our findings reveal that the caspase-3-induced ATM/p53/Cox-2/PGE_2_ signaling pathway participates in tumor repopulation in NSCLC. These results suggest that this pathway could be exploited to develop novel therapeutic strategies to counteract tumor recurrence during radiotherapy.

## RESULTS

### Radiations induce DNA damage, caspase-3 activation, and tumor repopulation in NSCLC cells

**Figure 1:**
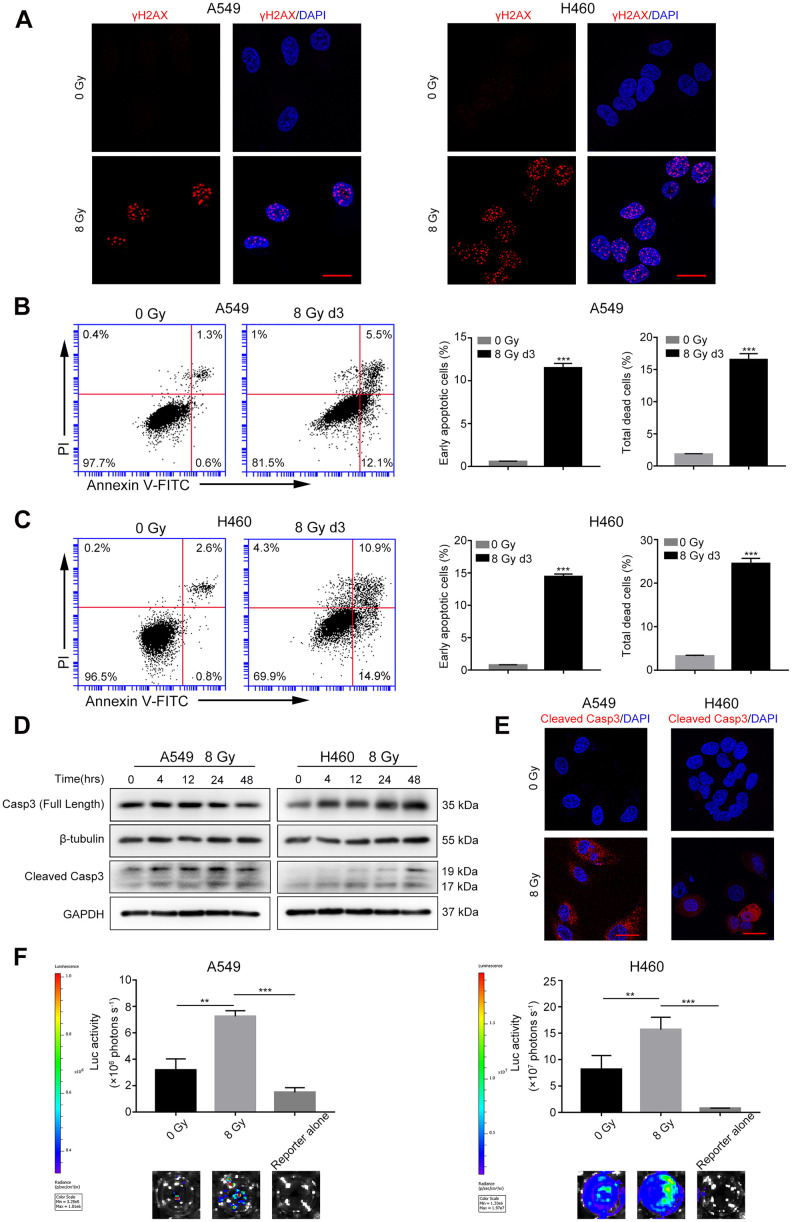
**Radiations induce DNA damage, caspase-3 activation, and tumor repopulation in NSCLC cells.** (**A**) Confocal images of immunostained A549 and H460 cells showing γH2AX foci following 8 Gy irradiation at 48 h. Scale bars: 25 μm. (**B**, **C**) The left panel shows flow cytometry analysis of A549 (**B**) and H460 (**C**) cell death after 0 Gy or 8 Gy irradiation on day 3. Apoptosis was monitored by Annexin V/propidium iodide (PI) double staining. The right panel shows quantitative analysis of early apoptosis and total cell death in 0 Gy- or 8 Gy-irradiated A549 (**B**) and H460 (**C**) cells (***p<0.001, Student’s *t* test, *n* = 3). (**D**) Cleaved caspase-3 induced by 8 Gy radiations was assayed by western blotting, and β-tubulin and glyceraldehyde 3-phosphate dehydrogenase (GAPDH) served as loading controls. (**E**) Representative confocal images of immunostained A549 and H460 cells showing cleaved caspase-3 following exposure to 8 Gy radiations on day 3. Scale bars: 25 μm. (**F**) The 8 Gy-irradiated NSCLC cells promoted the growth of living NSCLC reporter cells. The upper panel depicts luciferase activities showing the growth of A549 Fluc and H460 Fluc cells that were seeded alone or with 0 Gy- or 8 Gy-irradiated NSCLC cells. The lower panel shows the representative bioluminescence images (**p<0.01, ***p<0.001, one-way analysis of variance [ANOVA], *n* = 4).

We first performed a colony formation assay to find the optimal X-ray dose that induced tumor cell death. As shown in Supplementary Figure 1, the surviving fractions of A549 and H460 cells irradiated with 8 Gy X-ray were 0.043% ± 0.014% and 0.355% ± 0.018%, respectively. Thus, we selected 8 Gy dose to generate dying NSCLC cells. Phosphorylated histone H2AX (γH2AX) is a well-characterized marker of DSBs [19]. As shown in Figure 1A, compared with the control cells, the levels of γH2AX foci greatly increased in the 8 Gy-irradiated cells at 48 h after irradiation. Cell death was measured using Annexin V-fluorescein isothiocyanate (FITC) and propidium iodide (PI) double staining by flow cytometry. Compared with the control group, the percentage of early apoptotic cells (Annexin V-FITC positive and PI negative) and total dead cells (Annexin V-FITC positive) increased in both the 8 Gy-irradiated A549 and H460 groups on day 3 (Figure 1B, 1C). Because caspase-3 functions in the execution phase of apoptosis, we next used western blotting to determine whether it was activated following irradiation. We observed that 8 Gy irradiation generated cleaved caspase-3 (CC3) in a time-dependent manner in both A549 and H460 cells (Figure 1D). Moreover, immunofluorescence analysis revealed markedly enhanced expression of CC3 after 8 Gy irradiation (Figure 1E). These results demonstrate that 8 Gy irradiation induced DNA damage accompanied by cell death in NSCLC cells.

To investigate the effect of irradiated, dying NSCLC cells on living tumor cells, we conducted an *in vitro* repopulation experiment. The firefly luciferase (Fluc)-green fluorescent protein (GFP)-labeled cells were named Fluc cells (reporter cells). We observed that the luciferase activity of A549 Fluc or H460 Fluc cells linearly correlated with the cell numbers (Supplementary Figure 2); thus, we used luciferase assay to measure the proliferation of Fluc-GFP-labeled cells. Subsequent results demonstrated that 8 Gy-irradiated A549 feeder cells promoted the proliferation of A549 Fluc reporter cells as compared with A549 Fluc reporter cells growing on sham-irradiated feeder cells or no feeder cells (Figure 1F). Similarly, 8 Gy-irradiated H460 feeder cells exerted potent growth-stimulating effects on H460 Fluc reporter cells (Figure 1F).

### Casp3 KO attenuates the growth-promoting effect of dying NSCLC cells *in vitro*

**Figure 2:**
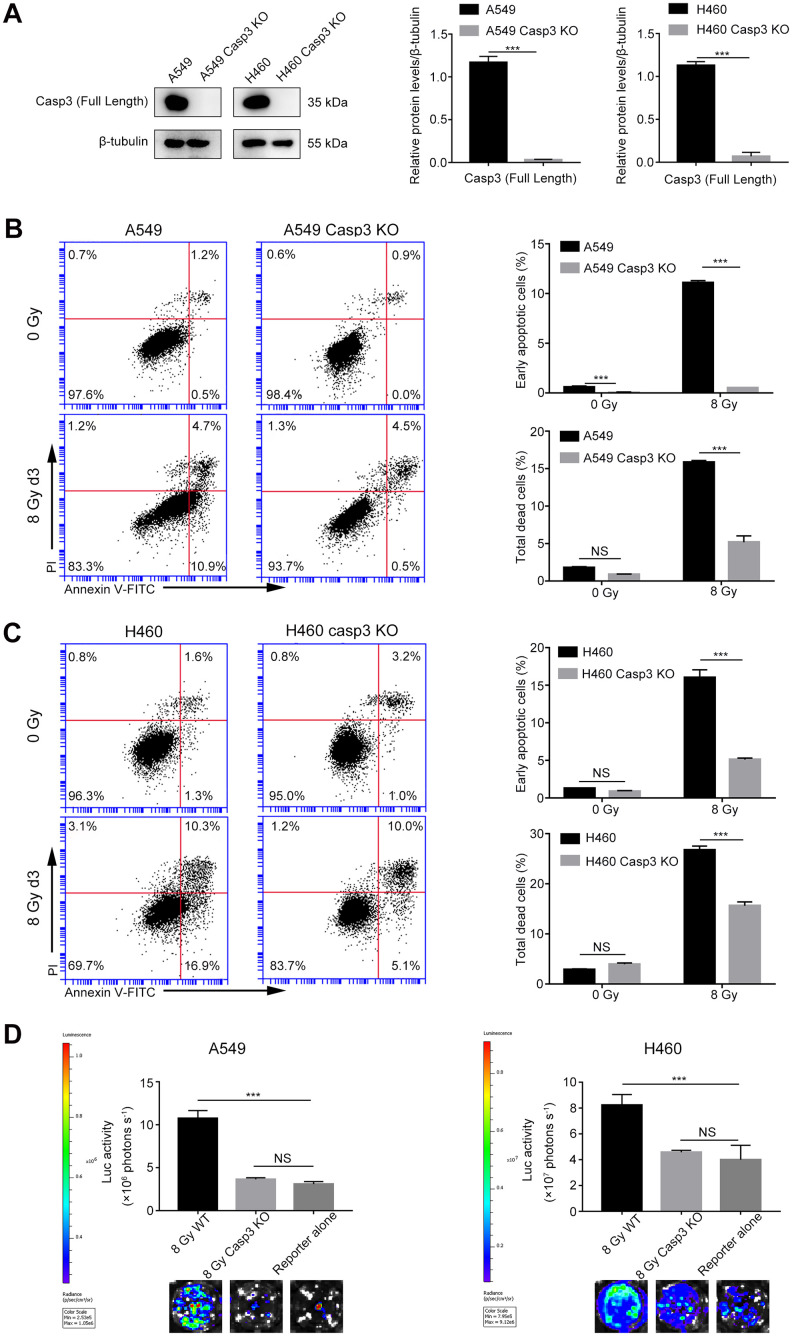
**Casp3 KO attenuates radiation-induced apoptosis and growth-promoting effect of dying NSCLC cells.** (**A**) Western blot analysis of the expression of caspase-3 in Casp3 KO A549 and H460 cells generated using the CRISPR/Cas9 system. β-tubulin was used as the loading control (***p<0.001, Student’s *t* test, *n* = 3). (**B**, **C**) The left panel shows the flow cytometry analysis of cell death in A549 and A549/Casp3 KO (**B**) and H460 and H460/Casp3 KO (**C**) cells following irradiation. Apoptotic cells were analyzed by Annexin V/propidium iodide (PI) double staining. The right panel shows the quantitative analysis of early apoptosis and total cell death in 0 Gy- or 8 Gy-irradiated control and A549/Casp3 KO (**B**) and H460/Casp3 KO (**C**) cells (***p<0.001, NS = not significant, Student’s *t* test, *n* = 3). (**D**) Casp3 KO significantly decreased the growth-promoting effect of 8 Gy-irradiated NSCLC cells on living NSCLC reporter cells. The upper panel depicts the luciferase activities showing the growth of A549 Fluc or H460 Fluc cells that were seeded with 8 Gy-irradiated wild-type or Casp3 KO cells or alone. The lower panel shows the representative bioluminescence images (***p<0.001, NS = not significant, one-way analysis of variance [ANOVA], *n* = 4).

We have previously reported a critical function of caspase-3 in breast and melanoma tumor cell repopulation [10, 20]. In the present study, we investigated whether caspase-3 exerted a growth-promoting effect of dying NSCLC cells. Using CRISPR/Cas9 technology, we generated A549 and H460 cells with genetic ablation of caspase-3 (Casp3 KO cells). First, we performed immunoblotting assays to assess the efficiency of Casp3 KO cells in different mutant single-cell clones (data not shown) and subsequently selected a clone with a sufficient Casp3 KO effect. As shown in Figure 2A, compared with the control A549 or H460 cells (wild-type), the levels of caspase-3 were reduced in selected Casp3 KO clones. Furthermore, we found that compared with the control group, the percentage of early apoptotic cells and total dead cells decreased in both 8 Gy-irradiated A549/Casp3 KO and H460/Casp3 KO groups on day 3 (Figure 2B, 2C). Using the *in vitro* repopulation model, we observed that 8 Gy-irradiated Casp3 KO feeder cells diminished the growth-stimulating effect of caspase-3 on both A549 and H460 living reporter cells (Figure 2D).

### Activated Cox-2/PGE_2_ signaling in dying cells promotes adjacent living tumor cell growth

**Figure 3:**
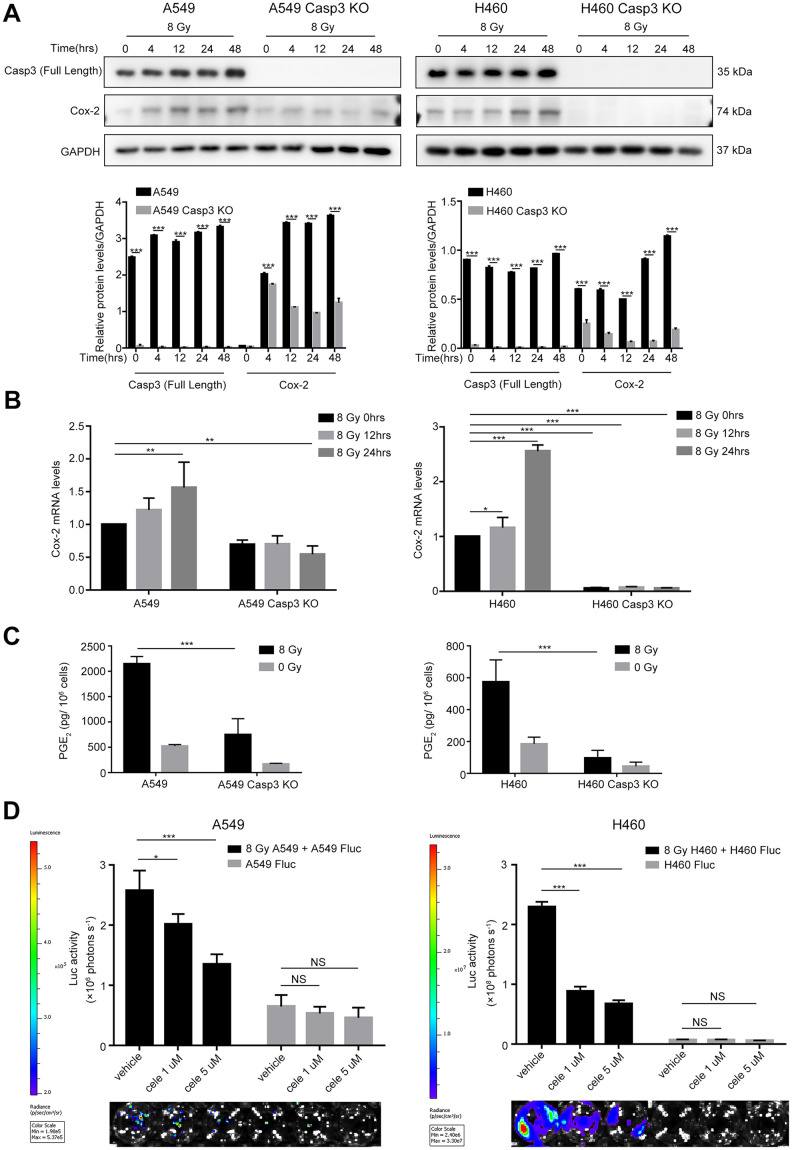
**Caspase-3-dependent PGE_2_ production in dying NSCLC cells induces tumor repopulation.** (**A**) Western blot analysis of Cox-2 levels at various time intervals after 8 Gy irradiation of wild-type and Casp3 KO NSCLC cells (***p<0.001, one-way analysis of variance [ANOVA], *n* = 3). (**B**) Quantitative polymerase chain reaction (qPCR) analysis of Cox-2 in wild-type and Casp3 KO NSCLC cells at indicated times after 8 Gy irradiation (*p<0.05, **p<0.01, ***p<0.001, one-way ANOVA, *n* = 3). (**C**) Levels of prostaglandin E_2_ (PGE_2_) in culture supernatants of wild-type and Casp3 KO NSCLC cells at 48 h after 8 Gy irradiation were measured using enzyme-linked immunosorbent assay (ELISA) (***p<0.001, one-way ANOVA, *n* = 3). (**D**) A selective Cox-2 inhibitor, celecoxib, abrogated the pro-proliferation effects of dying NSCLC cells on Fluc cells in a dose-dependent manner (*p<0.05, ***p<0.001, NS = not significant, one-way ANOVA, *n* = 4).

Because Cox-2 is involved in the production of bioactive lipid PGE_2_, and we previously identified PGE_2_ as a downstream effector of caspase-3 in tissue regeneration [21], angiogenesis [11], and breast tumor repopulation [10], we hypothesized that caspase-3 could promote PGE_2_ production by increasing Cox-2 expression in dying NSCLC cells. Western blotting and quantitative real-time polymerase chain reaction (qPCR) showed elevated expression and transcription of Cox-2 in both A549 and H460 cells after exposure to 8 Gy radiations in a time-dependent manner (Figure 3A, 3B). However, the expression and transcription of Cox-2 were markedly inhibited in Casp3 KO cells following 8 Gy irradiation (Figure 3A, 3B). We next analyzed the production of PGE_2_ in supernatants obtained from irradiated A549 and A549/Casp3 KO cells using enzyme-linked immunosorbent assay (ELISA). As shown in Figure 3C, the levels of PGE_2_ in 8 Gy-irradiated A549 cells on day 2 increased approximately fourfold as compared with those in non-irradiated A549 cells. However, the secretion of PGE_2_ was considerably lower in Casp3 KO cells with or without 8 Gy irradiation. Moreover, similar results were obtained in H460 and H460/Casp3 KO cells. To determine the function of PGE_2_ in regulating the growth-stimulating effect of dying NSCLC cells *in vitro*, we next studied whether the growth of living NSCLC cells was inhibited with the downregulation of Cox-2. Treatment with celecoxib (1 μM or 5 μM), a selective Cox-2 inhibitor, dramatically decreased the growth-stimulating effect of dying A549 or H460 feeder cells on A549 Fluc or H460 Fluc reporter cells in a dose-dependent manner (Figure 3D). In summary, these results demonstrate that PGE_2_ is involved in caspase-3-induced NSCLC cell repopulation after irradiation.

### Casp3 KO attenuates DDR, ATM/p53 signaling, and p53-induced Cox-2 expression in dying NSCLC cells

**Figure 4:**
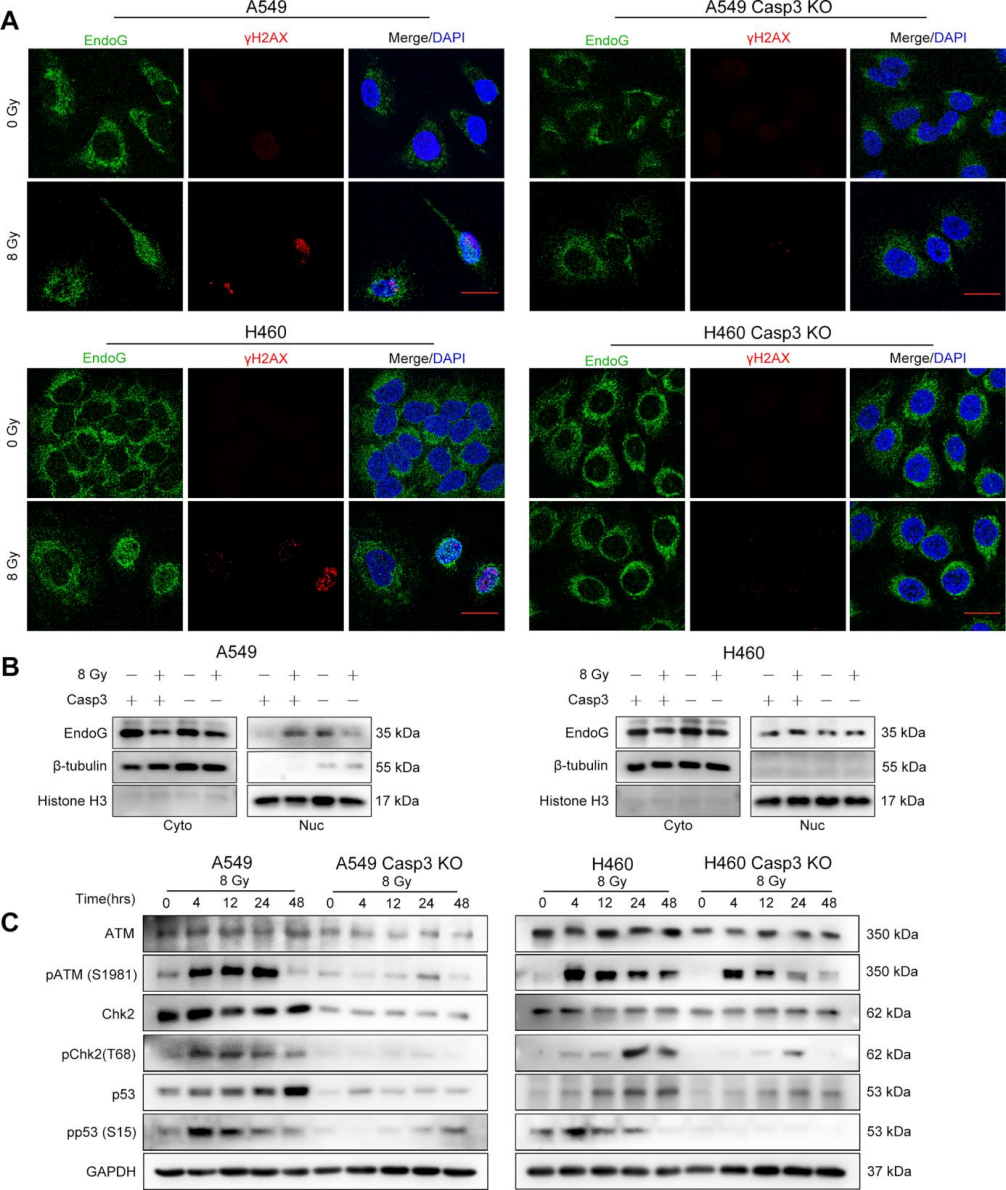
**Casp3 KO attenuates the DDR *via* ATM/p53 signaling in irradiated NSCLC cells.** (**A**) Immunofluorescence analysis of 8 Gy-irradiated wild-type and Casp3 KO NSCLC cells co-stained for EndoG and γH2AX foci at 48 h. Scale bars: 25 μm. (**B**) Western blot analysis of EndoG in the cytoplasmic and nuclear fractions of 8 Gy-irradiated wild-type and Casp3 KO NSCLC cells at 48 h. β-tubulin and Histone H3 were used as cytoplasmic and nuclear loading controls, respectively. (**C**) Levels of DNA damage response (DDR)-related proteins ATM, pATM (S1981), Chk2, pChk2 (T68), p53, and pp53 (S15) were measured by western blotting at indicated times after 8 Gy irradiation of wild-type and Casp3 KO NSCLC cells. GAPDH was used as the loading control.

We next studied the mechanisms by which caspase-3 enhanced the expression of Cox-2. During apoptosis, the mitochondrial protein endonuclease G (EndoG) migrates to the nucleus and cleaves the DNA [22]. The distribution of EndoG was determined through an immunofluorescence assay. As shown in Figure 4A, compared with the poor staining observed in the cytoplasmic regions of non-irradiated cells, radiotherapy increased the nuclear EndoG staining in NSCLC cells. Interestingly, caspase-3 activity regulates the cytoplasmic to nuclear migration of EndoG. The EndoG nuclear migration was suppressed in irradiated Casp3 KO cells, as evident from poor nuclear EndoG staining. Moreover, we found that irradiated wild-type cells with nuclear EndoG staining showed higher formation of γH2AX foci. However, the nuclear EndoG and γH2AX foci double staining was mostly absent in irradiated Casp3 KO cells. Next, we performed western blotting to examine the location of EndoG before and after irradiation of NSCLC cells (Figure 4B).

As ATM is a major sensor of DSBs [23], we assessed the protein levels of pATM (S1981) following irradiation. We observed robust ATM phosphorylation in parental A549 and H460 cells after irradiation. Interestingly, Casp3 KO reduced the levels of pATM and total ATM after irradiation (Figure 4C). Activated ATM phosphorylates several substrates, such as Chk2 and p53, thereby propagating the damage signal to numerous cellular pathways [23]. We next investigated whether activated ATM in A549 cells triggered the activation of Chk2 and p53. The levels of pChk2 (T68), p53, and pp53 (S15) were considerably higher after irradiation than in the control cells, whereas Casp3 KO reduced the levels of these proteins (Figure 4C). In addition, H460 cells showed similar results with or without Casp3 KO after irradiation (Figure 4C). These results suggest that caspase-3 induces DDR *via* ATM/p53 signaling.

**Figure 5:**
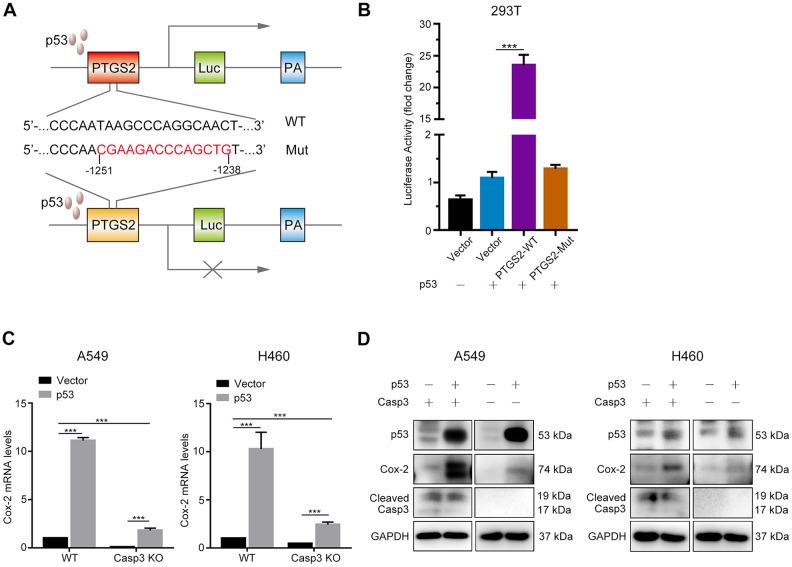
**p53 induces Cox-2 in NSCLC cells.** (**A**) Schematic representation of the luciferase reporter plasmid with the wild-type PTGS2 promoter sequence (PTGS2-WT) or mutant sequence (PTGS2-Mut). (**B**) A p53-dependent stimulation of PTGS2 promoter activity was demonstrated by luciferase assay. The 293T cells were co-transfected with p53 overexpression plasmid and PTGS2-WT plasmid, PTGS2-Mut plasmid, or vector alone. The pGMR-TK reporter was used as an internal transfection standard (***p<0.001, one-way analysis of variance [ANOVA], *n* = 3). (**C**, **D**) Quantitative polymerase chain reaction (qPCR) and western blot analysis showed that the mRNA and protein levels of Cox-2 were elevated by overexpression of p53 in wild-type and Casp3 KO NSCLC cells. Total RNA and proteins were extracted after transfection for 24 h and 48 h, respectively (***p<0.001, Student’s *t* test, *n* = 3).

It was previously demonstrated that the tumor suppressor p53 induced the expression of Cox-2 in response to DNA damage [24]. To elucidate whether p53 regulated the transcription of the *PTGS2* gene (encodes Cox-2), we first searched the JASPAR database (http://jaspar.genereg.net) to predict p53 binding sites in the PTGS2 promoter (Supplementary Figure 3). Next, we constructed a luciferase reporter plasmid encoding the PTGS2 promoter sequence (PTGS2-WT) or mutant sequence (PTGS2-Mut) in a region between −1251 bp and −1238 bp (Figure 5A). As shown in Figure 5B, p53-overexpressing cells exhibited higher luciferase activity in the PTGS2-WT group than in the control group. However, in the PTGS2-Mut group, the overexpression of p53 did not result in a major difference as compared with the controls. Next, we examined the transcript levels of Cox-2 following the overexpression of p53. As shown in Figure 5C, 24 h after transfection in A549 and H460 cells, the levels of Cox-2 transcript increased by more than 10-fold. We found a markedly less p53-induced expression of Cox-2 in Casp3 KO cell lines (Figure 5C). Further, western blotting results showed that the expression of Cox-2 in p53-overexpressing A549 and H460 cells was considerably higher than in p53-overexpressing Casp3 KO cells (Figure 5D). Based on these results, we conclude that p53, as a transcription factor, activates the expression of *PTGS2* in NSCLC.

### Caspase-3 knockout decreases tumorigenicity, and radiations activate the ATM/p53/Cox-2 axis *in vivo*

**Figure 6:**
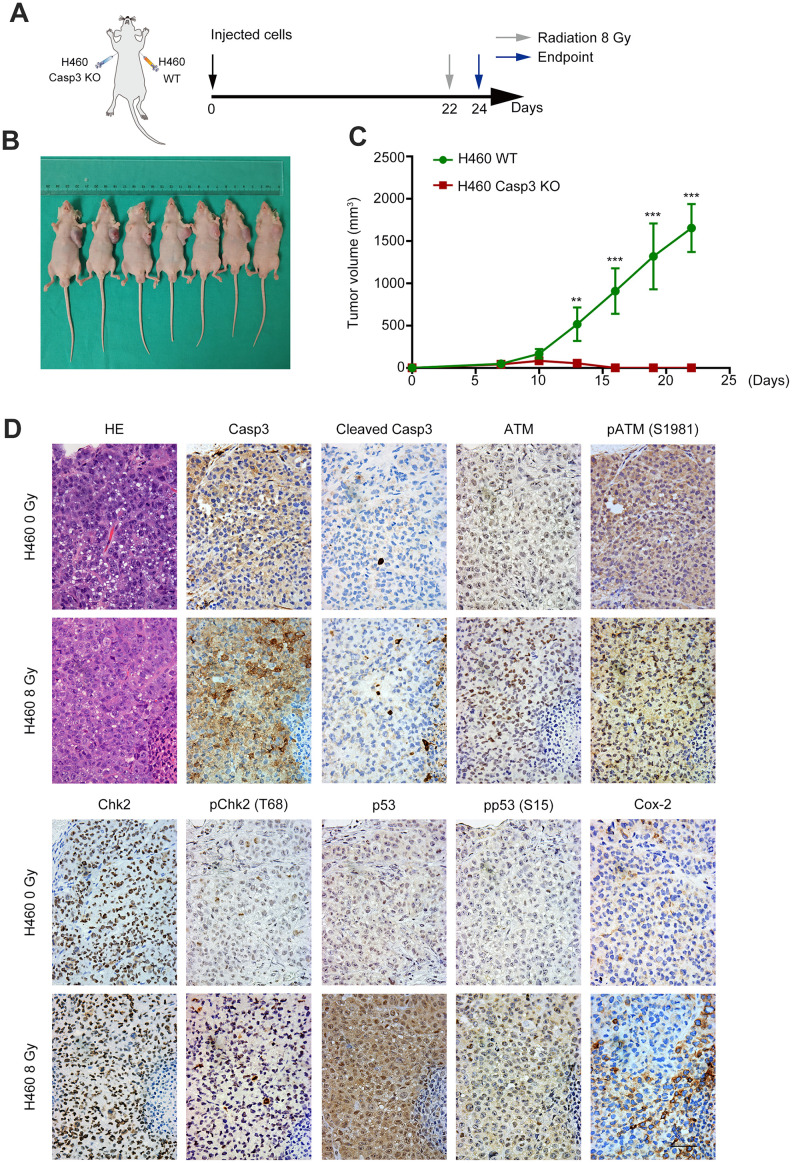
**Casp3 KO inhibits tumor formation, and radiations activate the ATM/p53/Cox-2 axis *in vivo*.** (**A**) Treatment scheme for nude mice. (**B**) Images of tumors obtained on day 22. (**C**) The tumor volume of xenografts was measured with calipers every 2 or 3 days (**p<0.01, ***p<0.001, Student’s *t* test, *n* = 7). (**D**) Representative photomicrographs of hematoxylin and eosin (H&E) and immunohistochemical staining of caspase-3, cleaved caspase-3, ATM, pATM (S1981), Chk2, pChk2 (T68), p53, pp53 (S15), and Cox-2 in tumor tissues. Scale bars: 50 μm.

**Figure 7:**
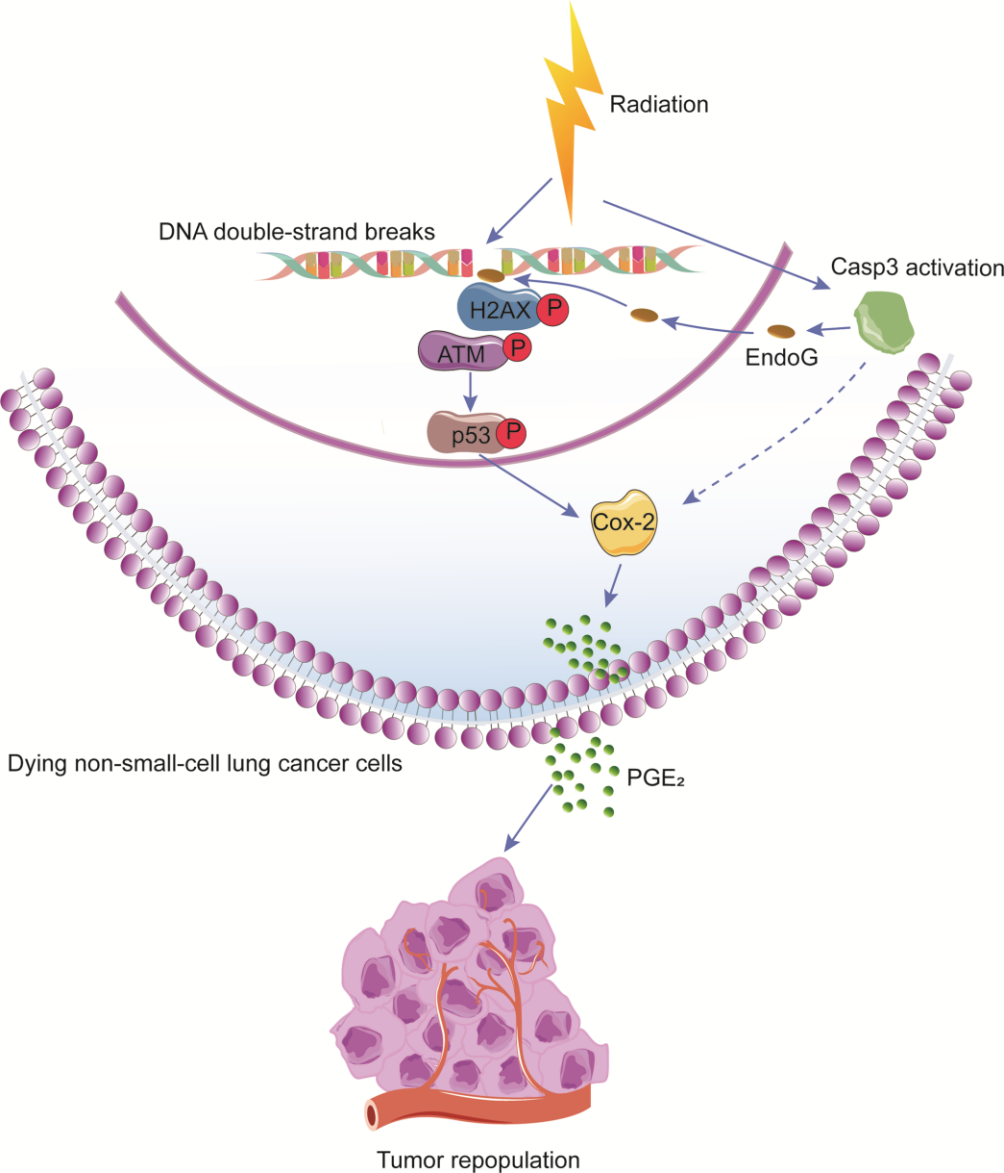
**Schematic illustration of the proposed mechanism of radiation-induced tumor repopulation in NSCLC.** Radiation-induced DNA double-strand breaks (DSBs) activate the DNA damage response (DDR) and caspase-3. Activated caspase-3 regulates the EndoG nuclear translocation and thus participates in the DDR by regulating ATM/p53 signaling, which activates the Cox-2/PGE_2_ axis in dying NSCLC cells, consequently enhancing the proliferation of living tumor cells.

To determine the effect of Casp3 KO on tumorigenicity *in vivo*, we subcutaneously inoculated wild-type and Casp3 KO H460 cells into nude mice. After 22 days, the volumes of the implanted tumors reached approximately 2000 mm^3^ in mice in the wild-type group (left armpit, 7/7). However, no tumor formation was observed in mice in the Casp3 KO group (right armpit, 0/7) (Figure 6A–6C). These data suggested that the knockout of caspase-3 inhibited tumorigenicity *in vivo*. Next, mice were randomly divided into two groups and exposed to either 0 Gy or 8 Gy radiations. Consistent with the *in vitro* results, immunohistochemistry showed enhanced levels of CC3 48 h after irradiation (Figure 6D). Moreover, a high number of pATM (S1981)-, pChk2 (T68)-, p53-, pp53 (S15)-, and Cox-2-positive cells were observed in mice in the 8 Gy radiation group (Figure 6D). The mRNA levels of p53 and Cox-2 were elevated in tumor tissues after irradiation (Supplementary Figure 4). Altogether, these results imply that the activation of caspase-3 and the ATM/p53 signaling pathway following irradiation induces the expression of Cox-2 (Figure 7).

## DISCUSSION

Radiotherapy treats cancer by triggering tumor cell death *via* apoptosis and/or necrosis [25, 26]. Caspase-3 is one of the core effector caspases responsible for apoptosis [27], and caspase-3 activity is widely used to evaluate the efficacy of anticancer therapeutics [28, 29]. In the present study, we demonstrated that caspase-3 coupled with DDR stimulated the proliferation of living tumor cells present adjacent to dying NSCLC cells, suggesting its involvement in tumor relapse following radiotherapy. A comparison of Casp3 KO cells with wild-type cells demonstrated that apoptotic caspase-3 induced tumor repopulation in NSCLC by 1) inducing DDR *via* activation of the ATM/p53 signaling pathway and by 2) activating the Cox-2/PGE_2_ axis *via* p53.

DDR is essential for the maintenance of genome integrity, and any defect in this repair process increases the predisposition to cancer [30]. DNA damage repair system comprises two main pathways: homologous recombination (HR) [31] and non-homologous end joining (NHEJ) [32]. In HR, cells use a homologous DNA sequence to guide accurate repair, whereas NHEJ involves ligating the broken ends after removing the damaged nucleotides at the end of DNA break sites. As a master regulator of DDR, ATM activates both HR- and NHEJ-mediated DNA repair pathways. Mutations in ATM have been implicated in NSCLC [33], and its loss has been reported as an early event in NSCLC carcinogenesis [34]. In addition, an ongoing phase I clinical trial (NCT03225105) is evaluating the efficiency of ATM inhibitor M3541 in combination with radiotherapy in patients with solid tumors [35]. Moreover, other DDR inhibitors in preclinical and clinical studies have been shown to improve antitumor activity in HR-deficient (HRD) tumors [36]. For example, olaparib, a pharmaceutical inhibitor of poly (ADP-ribose) polymerase (PARP), has been used successfully to treat BRCA-mutant ovarian [37], breast [38], prostate [39], and pancreatic cancers [40]. Although PARP inhibitors as single agents have been unsuccessful in treating BRCA-proficient cancers, including NSCLC [41], a recent study has reported that a combination of DNA methyltransferase inhibitors and PARP inhibitors enhanced the sensitivity of NSCLC cells to radiations [42].

Because DNA damage inducers, such as radiations, trigger apoptosis, the finding that apoptotic caspase-3 reversely promoted DSBs following irradiation in NSCLC cells was surprising [43]. The results of our study revealed a novel function of activated caspase-3, i.e., activation of DDR by promoting nuclear translocation of EndoG following irradiation. Our results are consistent with those of other studies. For instance, Liu et al. reported that a moderate radiation dose (≤ 6 Gy) sublethally activated caspase-3, causing DNA damage [44]. Similarly, Liu et al. found that activation of caspase-3 and nucleases resulted from spontaneous cytochrome C leakage, causing DNA damage and ATM activation, and leading to cancer stemness and tumorigenicity [13]. Another study reported that lethally activated caspase-3, in etoposide- or tumor necrosis factor (TNF)α-treated Hela cells, cleaved Cdc6 at D^290^/S and D^442^/G sites to activate ATM/ATR kinase and apoptosis [45]. Our findings demonstrated that lethally activated caspase-3 induced DDR following irradiation in an ATM/p53 pathway-dependent manner in NSCLC.

The Cox-2/PGE_2_ axis is involved in tumor initiation, progression, and recurrence [46]. In our study, compared with irradiated wild-type cells, caspase-3 knockout impaired the expression of Cox-2/PGE_2_ with a concurrent decrease in tumor repopulation. However, studies have reported controversial findings regarding the relationship between p53 and Cox-2 [24, 47–49]. In this study, the overexpression of p53 induced Cox-2, as revealed by a luciferase reporter assay, suggesting that Cox-2 acted as a transcriptional target of p53 in NSCLC. Our data showed that the absence of caspase-3 suppressed the expression of Cox-2, indicating a critical function of caspase-3 in the Cox-2 regulation. In addition, the protein levels of CC3 did not change after p53 overexpression in A549 and H460 cells (Figure 5D). This was consistent with the findings of previous studies, which demonstrated that the restoration of p53 in solid organ tumors primarily caused cell senescence than apoptosis [50, 51]. These results suggest that the Cox-2/PGE_2_ axis is a downstream target of the caspase-3-centered DDR pathway, which participates in radiation-induced tumor repopulation.

Although our study focuses on Cox-2/PGE_2_ as the downstream effector of caspase-3, radiation-induced dying NSCLC cells may also secrete additional growth-stimulating factors, such as vascular endothelial growth factor A (VEGF-A), to contribute to tumor relapse after radiotherapy [11, 52]. In addition, caspase-3 may modulate Cox-2 expression through other pathways [10, 11].

In summary, caspase-3 functions with DDR to induce tumor repopulation after radiotherapy in NSCLC, and the Cox-2/PGE_2_ axis controls the progression of NSCLC after radiotherapy. These molecules could provide promising therapeutic targets for NSCLC.

## MATERIALS AND METHODS

### Cell culture and irradiation

Human 293T cells and NSCLC cell lines H460 and A549 were purchased from the American Type Culture Collection. The 293T cells were cultured in Dulbecco’s Modified Eagle’s medium (DMEM), and H460 and A549 cells were cultured in Roswell Park Memorial Institute (RPMI) 1640 medium supplemented with 10% fetal bovine serum (FBS), and 100 units/mL penicillin and 100 μg/mL streptomycin (both from Thermo Fisher Scientific) at 37°C in a humidified incubator with 5% CO_2_. Cells or mice were irradiated in a cabinet X-ray generator (Faxitron) operated at 180 kVp and 10 mA with a dose rate of 3.0 Gy/min for the time required to apply a prescribed dose at room temperature.

### Lentivirus packaging and transduction

To construct lentivirus particles, the pLEX lentiviral system (Open BioSystems) was used to transduce genes into the target cells. The Fluc and GFP fusion gene was kindly provided by Prof. Chuan-Yuan Li. Fluc- and GFP-labeled cells were generated *via* lentivirus infection, as previously described [10]. Subsequently, the cells were cultured in RPMI 1640 medium supplemented with 10% FBS, and transfected cells were selected using 1 μg/mL puromycin for 2 weeks.

### Establishment of caspase-3 knockout cells

Casp3 KO A549 and H460 cells were established using the CRISPR/Cas9 genome editing system. The Casp3 KO lentivirus-based CRISPR plasmid [13, 53] (designated as the Casp3 KO plasmid) was also a kind gift from Prof. Li. The single guide RNA (sgRNA) sequence used to disrupt the *CASP3* gene was 5’-TAGTTAATAAAGGTATCCA-3’. This plasmid was packaged according to an established protocol [54]. A549 and H460 cells were seeded into a 6-well plate at a density of 5 × 10^5^ cells, and subsequently infected with the Casp3 KO plasmid-encoding lentivirus for 24 h and cultured in RPMI 1640 medium supplemented with 10% FBS. Forty-eight hours after infection, cells were selected in a culture medium containing 1 μg/mL puromycin for 2 weeks. Surviving cells were then trypsinized (Gibco) to obtain single cells that were seeded into 96-well plates at 1 cell per well. Clones derived from single cells were selected, and western blotting was used to identify the efficiency of genome editing after a clone expansion period. Clones with no detectable caspase-3 signal were selected for further study.

### Clonogenic assay

Cells were counted and seeded into 6-well plates. Next, the cells were exposed to different radiation doses (0, 2, 4, 6, or 8 Gy with 100 to 10,000 cells per well). After 10 to 14 days, cells were fixed with 4% paraformaldehyde (Sangon Biotech) and stained with crystal violet (Beyotime). Colonies containing more than 50 cells were scored under a Leica light microscope. The assay was performed in triplicate. The surviving fraction was calculated as previously described [55].

### Flow cytometry

Cells were treated with 8 Gy radiations for 72 h and subsequently stained with fluorescein isothiocyanate (FITC)–Annexin V and propidium iodide (PI) using a FITC Annexin V Apoptosis Detection Kit (BD Biosciences) following the manufacturer’s instructions. Apoptosis was measured on a BD Accuri C6 flow cytometer.

### Western blotting

Whole cell lysates were prepared in radioimmunoprecipitation assay (RIPA) buffer with protease and phosphatase inhibitors (Roche) at 4°C. Protein concentrations were determined using a bicinchoninic acid (BCA) protein assay kit (Thermo Scientific). Western blotting was performed as previously described [56]. Primary antibodies against β-tubulin, glyceraldehyde 3-phosphate dehydrogenase (GAPDH), caspase-3, CC3, Cox-2, ATM, pATM-S1981, pChk2-T68, pp53-S15 (#2128, #5174, #9662, #9664, #12282, #2873, #5883, #2197, #9286, respectively, Cell Signaling Technology), p53, EndoG, Histone H3 (#ab1101, #ab9647, #ab1791, respectively, Abcam), and Chk2 (#A19543, ABclonal) were used.

### Tumor repopulation model and bioluminescence imaging

In our *in vitro* repopulation model, 1 to 2.5 × 10^5^ irradiated cells (feeder cells) were co-cultured with a small number (200 or 500) of non-irradiated Fluc cells (reporter cells). During co-culturing, the culture medium was replaced with fresh RPMI 1640 medium containing 2% FBS every 2 days. After 6 to 10 days, the growth of Fluc cells was measured by bioluminescence imaging. D-Luciferin potassium salt (0.15 mg/mL; Synchem) was used as the bioluminescent substrate, and the bioluminescence was measured using the IVIS Lumin Series III imaging system (PerkinElmer).

### Quantitative real-time polymerase chain reaction

Total RNA was extracted from the cells using the RNA extracting reagent RNAiso Plus (#9109, Takara) and reverse transcribed into cDNA using the PrimeScrip RT Master Mix Kit (#RR036A, Takara). Quantitative real-time polymerase chain reaction (qPCR) was performed using the TB Green Premix Ex Taq Kit (#RR420A, Takara) according to the manufacturer’s instructions. The primers for *Cox-2* were 5’-GAAGTCCCTGAGCATCTACGG-3’ (forward) and 5’-CCTATCAGTATTAGCCTGCTTGTCT-3’ (reverse). The primers for *p53* were 5’-ACCTATGGAAACTACTTCCTGAAA-3’ (forward) and 5’-CTGGCATTCTGGGAGCTTCA-3’ (reverse). The primers for *GAPDH* were 5’-CCGGGAAACTGTGGCGTGATGG-3’ (forward) and 5’-AGGTGGAGGAGTGGGTGTCGCTGTT-3’ (reverse). GAPDH was used as the loading control. The qPCR procedure was performed under the following conditions: 30 s at 95°C, followed by 40 cycles of 5 s at 95°C and 30 s at 60°C. The results were obtained from three independent experiments. Differences in the relative expression were calculated using the 2^-ΔΔCT^ method.

### Transient transfection

For p53 overexpression, we transiently transfected the pcDNA3-p53 (WT) plasmid into cells using Lipofectamine 2000 reagent (Life Technologies) following the recommended protocol. The pcDNA3-p53 (WT) plasmid was synthesized by HarO Life, and the empty pcDNA3 plasmid (Invitrogen) was used as the control. Cells were incubated with Opti-MEM (Gibco) without FBS during transfections, and the transfection medium was replaced with RPMI 1640 medium after 6 h.

### PGE_2_ enzyme-linked immunosorbent assay

Cells were cultured in RPMI 1640 medium supplemented with 10% FBS and treated with 8 Gy radiations. Culture media were removed and replaced with fresh media containing 2% FBS for 16 h before the collection of supernatants at 48 h following irradiation. The levels of PGE_2_ in the supernatants were measured using the Prostaglandin E_2_ Express ELISA Kit (Cayman Chemical) according to the manufacturer’s instructions.

### Reagents

Celecoxib was purchased from Selleck (#S1261).

### Immunofluorescence staining

Cells were incubated with antibodies against γH2AX (#80312, Cell Signaling Technology), caspase-3 (#9662, Cell Signaling Technology), or EndoG (#ab9647, Abcam) overnight at 4°C, followed by incubation with an Alexa Fluor 488- or 594-conjugated secondary antibody (Proteintech) for 1 h at room temperature. Nuclei were counterstained with DAPI (Yeasen). Images were captured using a confocal laser scanning microscope (Leica Microsystems).

### Luciferase reporter assay

The upstream 2 kb promoter region of *PTGS2* (Cox-2) containing the potential p53 binding site was cloned into the GV238 (GeneChem) luciferase reporter vector (PTGS2-WT). Further, this region with a mutated p53 binding site was cloned into the same luciferase reporter vector (PTGS2-Mut). Next, 293T cells were co-transfected with the p53 overexpression (pcDNA3-p53) plasmid and PTGS2-WT plasmid, PTGS2-Mut plasmid, or empty vector for 24 h. Luciferase activity was determined using the dual luciferase reporter assay system (Promega), and the firefly luciferase activity was normalized to that of Renilla luciferase activity.

### Xenograft tumor model

All animal protocols were approved by the Shanghai General Hospital Institutional Animal Care and Use Committee and were conducted in accordance with the guidelines from the Directive 2010/63/EU of the European Parliament on the protection of animals used for scientific purposes. Five-week-old BALB/c mice were housed in specific pathogen-free (SPF) facilities with free access to normal chow and water. Wild-type or Casp3 KO H460 cells (5 × 10^6^ cells) were injected subcutaneously into the left and right armpit regions, respectively, of seven nude mice. The tumor volume (volume = length × width^2^/2) was determined using calipers every 2 to 3 days. When the mean tumor volumes reached approximately 2000 mm^3^, the mice were randomly divided into two groups: 0 Gy radiation (*n* = 3) and 8 Gy radiation (*n* = 4). Forty-eight hours after radiation treatment, all experimental mice were sacrificed, and tumor sections were collected for further pathologic examination.

### Hematoxylin and eosin staining and immunohistochemistry

Hematoxylin and eosin (H&E) staining and immunohistochemistry (IHC) were performed as previously described [57, 58]. Primary antibodies against caspase-3, CC3, Cox-2, ATM, pATM-S1981, pChk2-T68, pp53-S15 (#9662, #9664, #12282, #2873, #5883, #2197, #9286, respectively, Cell Signaling Technology), p53 (#ab1101, Abcam), and Chk2 (#A19543, ABclonal) were used. The sections were incubated with horseradish peroxidase-conjugated secondary antibody (EnVision III Detection System; GK500705; GeneTech), and counterstained with hematoxylin before visualized using a Leica light microscope.

### Statistical analysis

All data are expressed as mean ± standard error (SE). Statistical analysis was performed using unpaired Student’s *t* test or one-way analysis of variance (ANOVA) with SPSS version 18.0. A *P*-value < 0.05 was considered significant.

## Supplementary Material

Supplementary Figures
