# Supplementary material for: Caspase-3 knockout attenuates radiation-induced tumor repopulation via impairing the ATM/p53/Cox-2/PGE2 pathway in non-small cell lung cancer
Source: Aging (Albany NY). 2020 Nov 7;12(21):21758–76. doi: 10.18632/aging.103984 (PMC7695367; doi:10.18632/aging.103984)
Supplement: Supplementary Figures [file aging-12-103984-s001..pdf]

SUPPLEMENTARY FIGURES

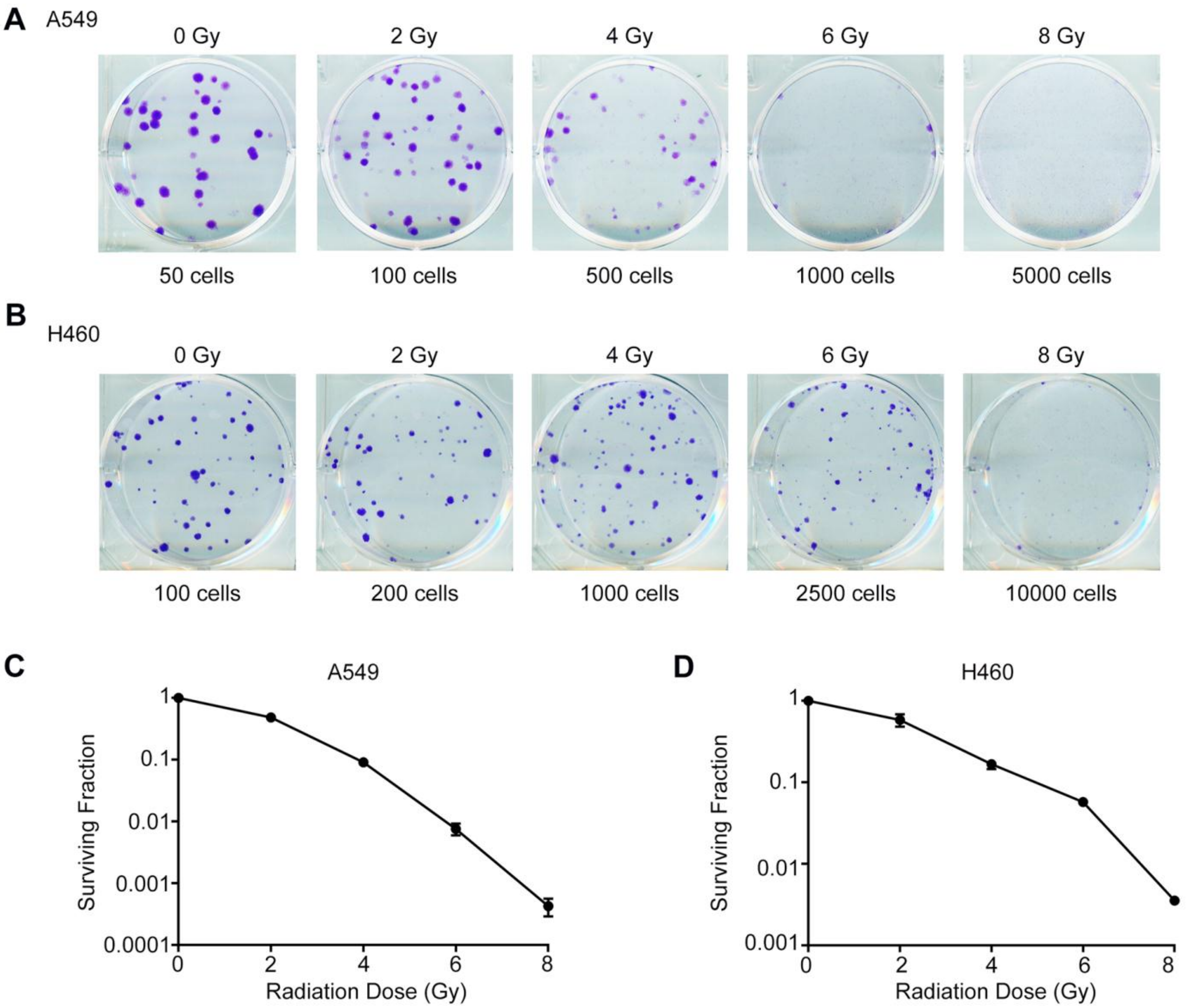

Supplementary Figure 1. Clonogenic cell survival assays in A549 and H460 cell lines after exposure to ionizing radiations. (**A**, **B**) The corresponding images of surviving colonies (>50 cells per colony) in A549 and H460 cells. (**C**, **D**) Surviving fractions of irradiated A549 and H460 cells, n = 3.

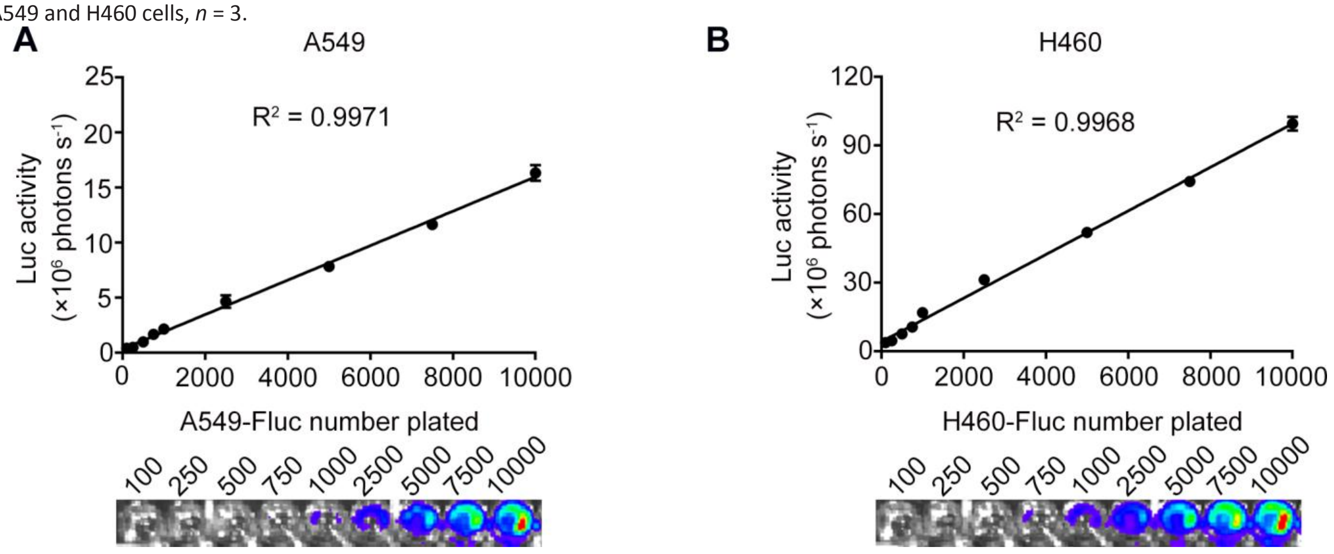

Supplementary Figure 2. A linear correlation was observed between the luciferase activity of A549 Fluc or H460 Fluc cells with seeded cell numbers. (**A**, **B**) Upper panel shows luciferase activities of different numbers of A549 Fluc and H460 Fluc cells. Lower panel shows the representative bioluminescence images, *n* = 4.

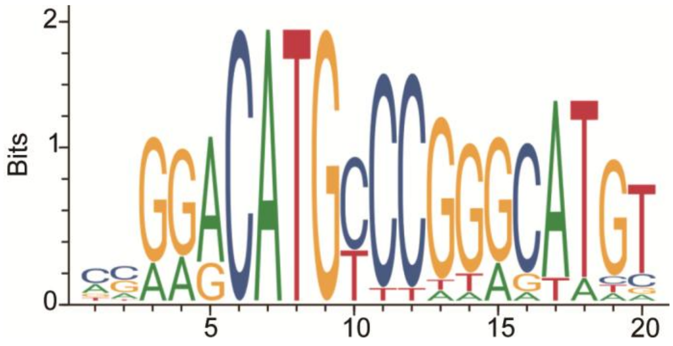

Supplementary Figure 3. The JASPAR database shows the potential p53 binding sites in the promoter region of *PTGS2*.

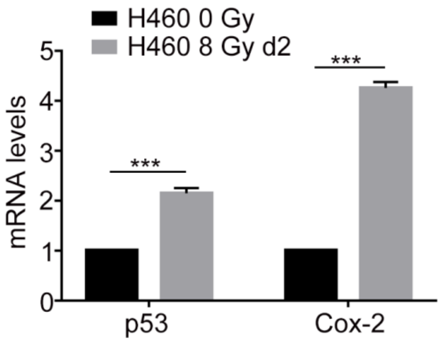

Supplementary Figure 4. The mRNA levels of p53 and Cox-2 in a xenograft mouse model after exposure to 8 Gy radiations (****p*<0.001, Student's *t* test, *n* = 3).**
